# Asymmetric polar localization dynamics of the serine chemoreceptor protein Tsr in Escherichia coli

**DOI:** 10.1371/journal.pone.0195887

**Published:** 2018-05-17

**Authors:** Dongmyung Oh, Yang Yu, Hochan Lee, Jae-Hyung Jeon, Barry L. Wanner, Ken Ritchie

**Affiliations:** 1 Department of Physics and Astronomy, Purdue University, West Lafayette, IN, United States of America; 2 Department of Biological Sciences, Purdue University, West Lafayette, IN, United States of America; 3 Department of Physics, Pohang University of Science and Technology, Pohang, South Korea; 4 Department of Microbiology and Immunobiology, Harvard Medical School, Boston, MA, United States of America; Centre National de la Recherche Scientifique, Aix-Marseille Université, FRANCE

## Abstract

The spatial location of proteins in living cells can be critical for their function. For example, the *E*. *coli* chemotaxis machinery is localized to the cell poles. Here we describe the polar localization of the serine chemoreceptor Tsr using a strain synthesizing a fluorescent Tsr-Venus fusion at a low level from a single-copy chromosomal construct. Using photobleaching and imaging during recovery by new synthesis, we observed distinct asymmetry between a bright (old) pole and a dim (new) pole. The old pole was shown to be a more stable cluster and to recover after photobleaching faster, which is consistent with the hypothesis that newly synthesized Tsr proteins are inserted directly at or near the old pole. The new pole was shown to be a less stable cluster and to exchange proteins freely with highly mobile Tsr-Venus proteins diffusing in the membrane. We propose that the new pole arises from molecules escaping from the old pole and diffusing to the new pole where a more stable cluster forms over time. Our localization imaging data support a model in which a nascent new pole forms prior to stable cluster formation.

## Introduction

About 4% of *Escherichia coli* membrane proteins are localized at the cell poles, which is critical for their function [1]. The polar localization of chemoreceptors, like the serine chemoreceptor Tsr, is required sensing environmental signals [1]. Tsr is a highly abundant transmembrane methyl-accepting chemotaxis protein (MCP) and is stably localized at the poles in variably sized clusters [2], which are necessary for collaborative signalling [3]. Tsr is one of five MCPs that form heterotrimeric membrane complexes at the poles [4]. However, the polar distribution of Tsr is asymmetric and changes during the cell cycle. Such asymmetry may be crucial in chemotaxis, chromosome segregation, and motility [3, 5]. One way polar localization is thought to be achieved depends on the geometrical curvature of the cell and segregation of chromosomal DNA to the polar regions [5].

The problem of polar localization has been previously investigated using single molecule techniques which provide direct imaging of the trajectories of individual molecules in living cells. Deich et al. [6] showed that the membrane-bound histidine kinase PleC localizes to the flagellar pole in motile swarmer cells of *Caulobacter crescentus* during asymmetric cell division. The mobile fraction of newly synthesized PleC molecules are homogeneously distributed in the cytoplasmic (inner) membrane and show no active, direct transport to re-localize during development from the motile swarmer cell to the pre-divisional cell. Similar results were observed for the *C*. *crescentus* chromosome anchor protein PopZ [7, 8]. These results support a model in which protein redistribution arises from random diffusion and capture, as proposed by Rudner et al. [9] and Shapiro et al. [10]

Other mechanisms of polar localization also exist. Targeting of the outer membrane virulence protein IcsA to the pole, which is required for unidirectional actin-based movement of *Shigella flexneria* in the host cell [11], is generated by its delivery exclusively at one pole. A polar gradient of IcsA is generated by lateral diffusion from the pole through the outer membrane. Similar observations were also seen in *E*. *coli* as well as more distantly related *Yersinia pseadotuberculosis* [12], suggesting that the mechanism of polar targeting of these outer membrane proteins is conserved. Jain et al. [13] extended these findings to the large autotransporter family of virulence proteins. Chromosome segregation during cell division in *E*. *coli* and related bacteria depends on separation of the nucleoids to polar regions. Woldringh et al. [14] proposed that this is acheived by transient constraining of DNA segments by coupled transcription-translation-secretion (“transertion”) of membrane proteins and a bias in the creation of transertion regions when chromosomes are replicated bidirectionally and newly replicated neighboring genes for membrane proteins are expressed.

Considering the localization of chemoreceptors in *E*. *coli*, recent publications have shown the importance of the Tol-Pal complex [15], nucliod-exclusion [16] and membrane curvature [17]. Shiomi et al. [18] showed that the aspartate chemosensor Tar first appeared within the cylindrical portion of the cell and was distributed in a helical pattern that colocalized with the Sec translocation machinery but not with the helical cytoskeletal protein MreB. They found Tar localized to the poles 80 minutes after induction. Thiem et al. [19] showed through fluorescently labeling CheR, which binds to the C-terminal of Tsr and Tar, that clusters of chemoreceptors occur at regular intervals along the cell body marking future cell division sites. Also using fluorescently labeled CheR, Santos et al. [15] showed that deletion of the Tol-Pal trans-envelope complex severely disrupted the polar localization of chemoreceptor clusters. Neeli-Venkata et al. [16] proposed a model where nucleoid exclusion coupled with diffusion-and-capture by the Tol-Pal complex fit their results on Tsr localization. Further, membrane curvature at the poles has been shown to assist in localization of large clusters of chemoreceptors [17, 20]. It has been shown elsewhere that Tsr accumulates at the poles linearly over time [21]. The greater accumulation of Tsr at the old pole relative to the new pole was used to discriminate the two poles in this study. Here we examined how Tsr is localized to the poles. We coupled ensemble and single molecule quantitative imaging methods to study the asymmetry of the poles in *E*. *coli*.

We recently found that the majority of newly synthesized Tsr accumulate at the old poles of *E*. *coli* and diffuse at different rates at the polar and cylindrical surface regions [22]. We also showed that there is no active transport for Tsr mobility. Single pole photobleach/recovery experiments show that newly emerging molecules are first aggregated at the old pole. Direct fluorescence imaging implies that the new pole matures through capture of molecules escaping the old pole and diffusing to the new pole region. Our results are consistent with the model that newly synthesized Tsr is first inserted at or near the old pole, where Tsr is captured by existing chemotaxis complexes, but can escape and freely diffuse throughout the cell membrane and captured by small or unstable seeds of Tsr (or other chemotaxis complexes) clusters at the new pole.

## Results

Experiments were carried out using cells attached to glass slides which permitted examining individual cells over time. By observing numerous cells over 5 min growth periods, we quantified the positions of individual Tsr-Venus molecules within single cells. In these experiments, we bleached all Tsr-Venus fluorescence in living cells at time zero and again at 1-min intervals after imaging newly observed Tsr-Venus molecules. Cells were sheltered from illumination between observations to allow for newly synthesized and maturing Tsr-Venus molecules appear without photobleaching.

We found through direct single molecule counting, a total of 2356 new fluorescent spots among 1956 cells were observed over a 5 minute time scale, with 630 cells displaying more than one event, implying an upper bound for the protein production rate of 0.24 proteins per cell per min. Further, spots were not homogeneously distributed; rather, 83% were localized near poles, defined as a sphere 0.5 μm in radius, located within 0.5 μm from a cell end. As expected, the synthesis rate of Tsr-Venus is low (0.24 proteins/min measured over 1956 cells) and newly synthesized Tsr-Venus was first observed mostly near the poles, which is where MCP chemotaxis clusters are located (see Table A in S1 File).

**Fig 1:**
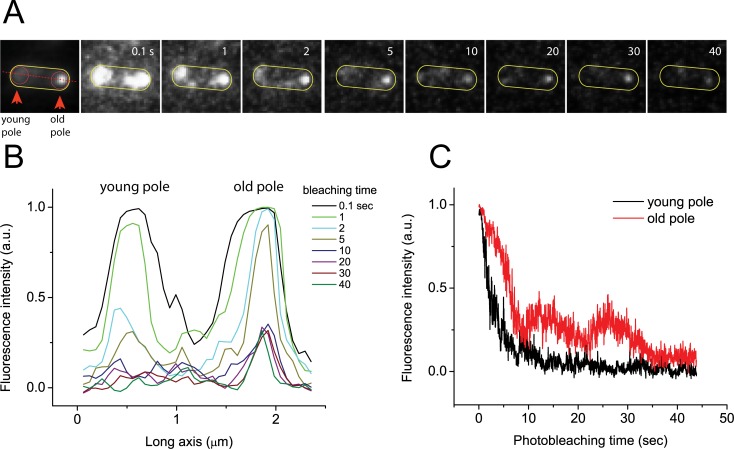
Whole cell photobleaching. A) Integrated image over time and film strip from video of the whole cell photobleaching of an individual cell. B) Intensity profiles over time from film strip in A along red dotted line in the integrated image. C) Integrated intensity in each polar region (circles in A) over time.

To investigate the photostability of the poles, we performed whole cell photobleaching. Fig 1A shows individual image frames extracted from a typical movie (out of ~100 cells imaged) (S1 Movie) showing photobleaching of both poles under continuous illumination. The left panel is an integrated image of S1 Movie (1316 frames, at 30 fps), which shows one pole is significantly brighter than the other when viewed as a single, long exposure image with much enhanced signal-to-noise ratio. Here we will refer to the bright pole from the integrated image as the “old pole” and the dimmer pole as the “young pole” [21]. The image sequences show young poles bleach more quickly than the old poles throughout. Fig 1B shows the intensity profile of a linear cross section along the long axis of the cell (red dotted line in the left panel of Fig 1A) as a function of bleaching time. Fig 1C shows the total intensity of young and old poles (circled areas in left panel of Fig 1A). While photobleaching of young poles follows an expected exponential decay, photobleaching of old poles deviates from such a decay rate. The fluorescence intensity at old poles was reduced only 30% within 5 sec while intensity of young poles dropped 80% (Fig 1C). The differential behaviour of photobleaching of old and young poles (Fig A in S1 File, S2 Movie) may result from different dwell times or the acquisition of newly synthesized Tsr-Venus proteins at old poles, which is consistent with results above.

**Fig 2:**
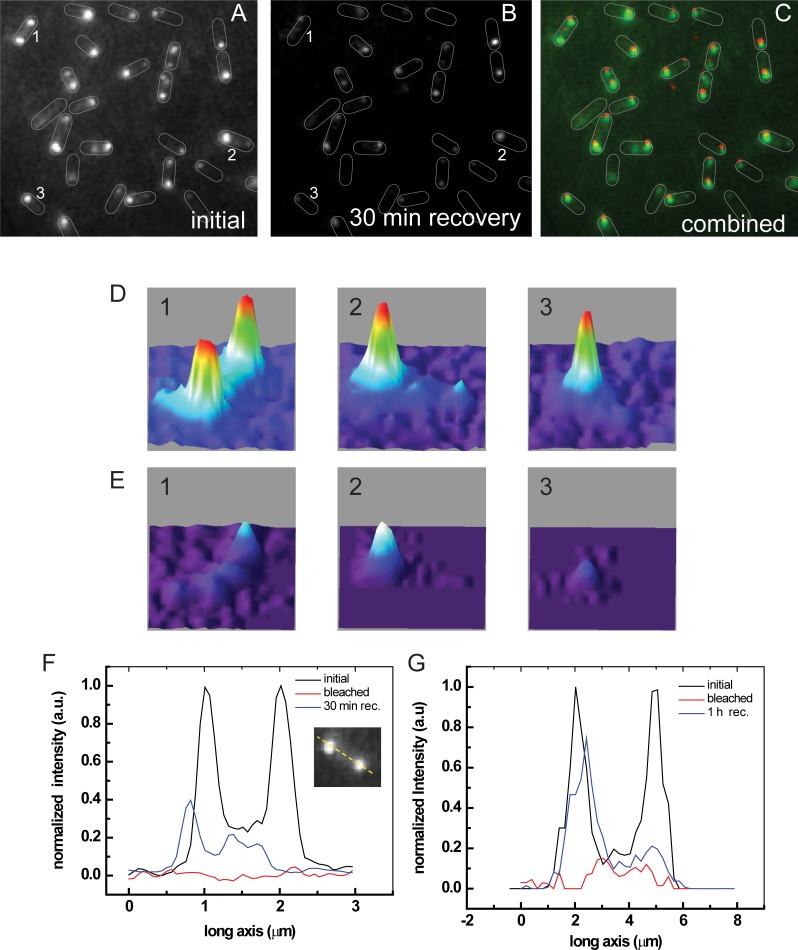
Whole cell recovery after photobleaching. A) Fluorescence before photobleaching (pre-bleach). B) Fluorescence after 30 min recovery (post recovery). C) Combined image (Green: pre-bleach, Red: post recovery). Overlaid cell boundaries have been added to guide the eye. D) Initial fluorescence of cells 1, 2, and 3 in Panels A. E) Fluorescence of cells 1, 2, and 3 after photobleaching and 30 min. recovery showing fast recovery of one pole and slow recovery of other pole. F) and G) Fluorescence profiles showing asymmetric recovery after 30 min and 1 hour, respectively.

Fig 2 shows fluorescence of Tsr-Venus in cells within a field showing many cells. Fig 2A is a fluorescence image of cells cultured overnight, which shows bright spots at one or both poles of individual cells, indicating a long-term uni- or bi-polar localization of Tsr-Venus. Fig 2D shows the fluorescence intensities of the poles in cells 1, 2, and 3 (in Fig 2A and 2B), showing variation in fluorescence from pole to pole in single cells and between different cells within the field. The poles in cell 1 show similar fluorescence, one pole is brighter than the other in cell 2, and only one pole is brighter than background in cell 3. Because cell growth was not synchronized, the cell-to-cell variation in fluorescence may result from cell cycle-dependence on Tsr-Venus localization or other causes.

To examine where newly synthesized proteins are eventually localized, cells in Fig 2A were photobleached and allowed to recover for 30 min and 1 hour before imaging. Fig 2B shows fluorescence after 30 min recovery; Fig 2E shows fluorescence of cells 1, 2, and 3 after 30 min recovery. Fig 2C is an overlay of images for the initial and recovered fluorescence (Green: pre-bleach, Red: post recovery). On average, cells recovered about 33% of their total initial fluorescence intensity through new synthesis and maturation of Tsr-Venus. Comparing the recovery pattern in each cell, we found one pole consistently recovered more quickly than the other. If one pole was brighter than the other before bleaching (e. g., cells 2 and 3), the brighter pole recovered more fully. When the poles were equally intense (e. g., cell 1), the fluorescence intensity recovered fully only at one pole and increased over time, as shown in Fig 2F and 2G after 30 min and 1 hr recoveries, respectively.

To test fluorescence intensity recovery at the poles individually, we shuttered illumination of one pole, photobleached the other, and monitored the fluorescence reduction of the sheltered pole. Loss of fluorescence from the sheltered area occurs when the number of escaping molecules exceed the production of new Tsr-Venus molecules. For cells with one brighter pole, sheltering the brighter one and photobleaching the other led to an average of 25% (n = 21 cells) loss of fluoresecence intensity of the sheltered pole. In contrast, sheltering the dimmer pole and photobleaching the brighter one led to an average of 90% (n = 50) loss of fluoresecence intensity of the sheltered pole. These results imply that Tsr-Venus molecules at the dimmer pole are more loosely held and can easily escape. These experiments underline the asymmetry of Tsr complexes of the poles (S3 Movie).

**Fig 3:**
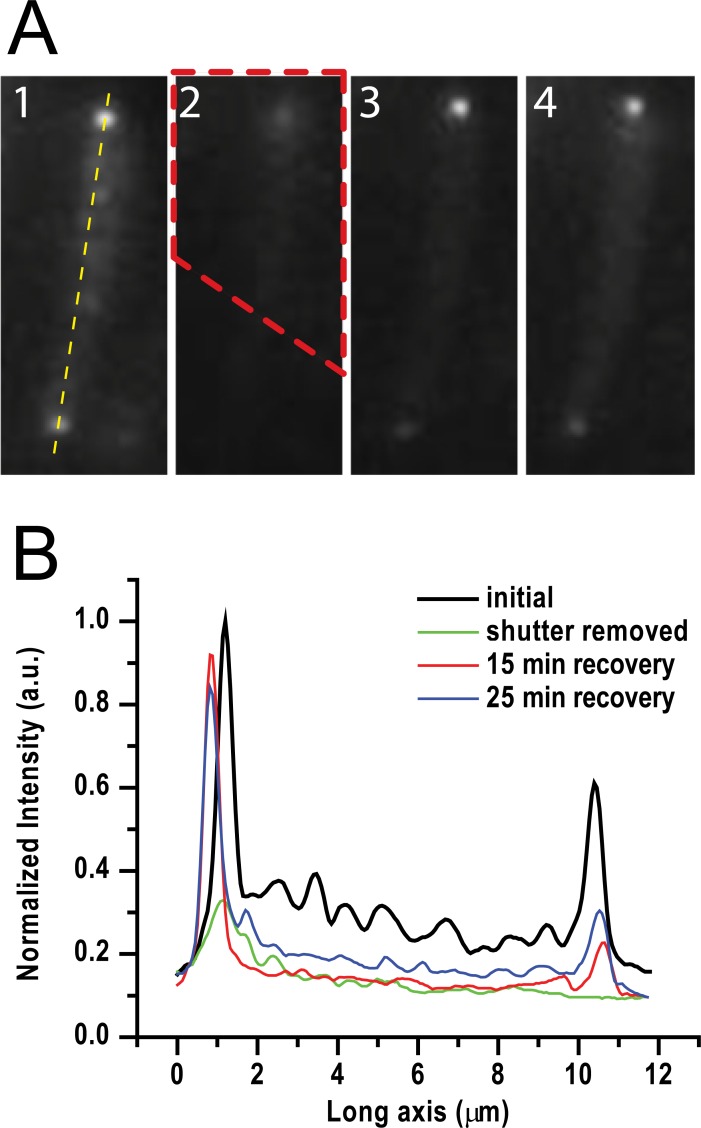
Single pole photobleach and recovery. A) 1. Initial fluorescence, 2. Image immediately after shutter (red dashed area) removed, 3. Image after 15 min recovery, 4. Image after 25 min recovery. B) Intensity profiles along yellow dashed line in A1 showing fast recovery of the brighter, sheltered pole.

To further examine the asymmetry of Tsr-Venus at the poles, we carried out similar experiments except in the presence of subinhibitory chloramphenicol (20 μg/ml, final), which blocks cell division without inhibiting protein synthesis [23], leading to elongated cells of ca. 10 μm in length. Fig 3A illustrates recovery of fluorescence intensity of an elongated cell with poles of similar brightness. Panel 1 shows the pre-beached fluorescence image, where the dashed yellow line marks the long axis of the cell; panel 2 shows the image after removal of the shutter, where the dashed red line outlines the area shuttered; panels 3 and 4 show the fluorescence images after 15 min and 25 min recovery, respectively. Fig 3B shows the fluorescence intensities along the long axis of the cell in panels 1 to 4. Although signal reduction was seen for both the sheltered and unsheltered poles (green line), the signal recovered faster at the sheltered pole than at the bleached pole (red and blue lines in Fig 3B), though the bleached pole does slowly recover.

**Fig 4:**
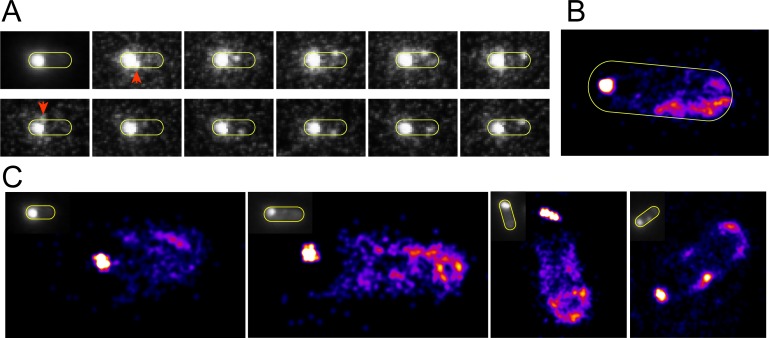
Localization imaging of the nascent new pole. A) Representative integrated image and filmstrip showing escape of molecules from the brighter old pole and diffusion toward the new pole region. B-C) Localization images of the propensity of Tsr-Venus to reside in the entire cell. The sharp bright dots show an artefactually small localization of the bright stable old pole cluster. At the opposite poles, the areas where the new pole forms show no indication of cluster formational though bulk fluorescence imaging (insets).

We also performed direct observation of Tsr-Venus dynamics near the poles under continuous illumination. Fig 4B (S3 Movie) shows a cell with one bright pole from which single Tsr-Venus proteins or small protein clusters containing Tsr-Venus escape and diffuse to the dark pole. The first panel of Fig 4A shows an integrated fluorescence image, in which significant fluorescence signal are seen only in areas of the cell with stable clusters containing Tsr-Venus and mobile fluorescent signals are averaged out. Subsequent panels show movie frames in which small clusters of Tsr-Venus proteins, marked with red arrows, diffuse (“escape”) from a bright pole to the other pole. S3 Movie also shows Tsr-Venus entering the bright pole. Escaping Tsr-Venus were also seen to reside longer in areas near the poles than in central regions of the cell.

We adopted the analysis techniques of spt-PALM [24] to examine the propensity of mobile Tsr-Venus molecules to reside near the poles. We obtained movies ca. 1 min length under continuous illumination and reconstituted localization images using a point spread function (PSF) with 1 grey value and 16 nm FWHM on measured trajectories. Fig 4B is a reconstituted image with 2,798 PSFs and 1316 image frames showing several dim clusters near the young pole. The strong, sharp cluster near the bright pole is from the stable cluster at that end. The extent of distribution of the bright cluster is severely reduced due to applying spt-PALM to the extended cluster of molecules. Fig 4C shows similar spt-PALM images using n = 1493, n = 2790, n = 2446, and n = 3714 PSFs from S3, S4, S5 and S6 Movies, respectively. Together the spt-PALM images provide further support that the maturing young pole is populated by diffusing Tsr-Venus molecules escaping from the more stable bright pole.

## Discussion

Our results show a distinct asymmetry of the cell poles both in terms of stability and recovery. By using Tsr-Venus, we specifically showed: a) the bright (old) pole photobleaches more slowly than the dim (new) pole when photobleaching the whole cell (Fig 1); b) the old pole recovers fluorescence intensisty faster after whole cell photobleaching; c) both poles exchange Tsr-Venus with diffusing molecules in the membrane, however the old pole is more stable than the young pole (Figs 2 and 3); and d) localization analysis shows areas of the new pole where the new polar cluster forms (Fig 4).

Results from single pole photobleaching and recovery show the relative stability of the two poles. When both poles show similar intensities, sheltering the new pole from photobleaching shows substantial reduction of fluorescence of the new pole, implying that molecules localized near the new pole easily escape. Conversely, sheltering the old pole reveals much less reduction of fluorescence of the old pole, implying that the old pole is much more stable and/or rapidly accumulates newly synthesized Tsr-Venus after bleaching. We also observed that the old pole recovered fluorescence much faster than the new pole region after bleaching either pole singly.

We previously showed that Tsr-Venus molecules in the inner membrane freely diffuse on 5 ms time scales [22]. We also found that the majority are trapped at the poles within nested compartments of 170 nm and 290 nm length scales. A non-Gaussianity test of individual Tsr-Venus trajectories showed that Tsr-Venus was constrained by these compartments at time scales longer than 5 ms. Notably, we found no evidence that Tsr-Venus is stably immobilized to any immobile structure at the poles, rather Tsr-Venus molecules are restricted from escape (see S1 File) which is consistent with recently found mechanisms for corralling chemoreceptors into the polar region (such as the Tol-Pal complex [15] and membrane curvature effects [17]). A small fraction (7%) of molecules was found diffuse freely in the membrane with a diffusion coefficient of 0.4 μm^2^/s. Presumably, these molecules are the ones that escape and are free to explore the entire cell. They may originate from the relatively stable old pole or unstable newly forming new pole.

By applying single molecule localization analysis techniques to images of the freely diffusing Tsr-Venus molecules, we obtained trajectories showing a propensity of Tsr-Venus to localize near the new pole, further underlining the relative stability of the two poles (S1 File). Together these findings are consistent with a transertion-diffusion-capturing mechanism for pole maturity combining the previously proposed transertion mechanism [14] with diffusion-capturing mechanisms[6, 9, 15–17] for the polar localization of Tsr at the poles.

Accordingly, we propose that newly synthesized Tsr preferentially accumulates near the old pole by interacting with relatively stable chemotaxis complexes containing other proteins of the chemotaxis machinery at that pole. The new pole is populated by Tsr molecules (or small clusters) that escape the old pole and diffuse through the membrane to the newly forming pole. Over time, accumulation of Tsr into clusters containing other chemotaxis proteins at the new pole forms stable chemotaxis complexes that matures into stable old pole structures during cell division.

## Materials and methods

### Cells

*E*. *coli* K-12 BW36931 has a single copy of the CRIM plasmid[25] pYY37 (*attλ kan phoAp*-*tsr*-*venus*) in BW36010 (MG1655 Φ(*lacZp4105*(UV5)-*lacY*)*638*(DE*lacZ*) *rph*^+^ DE(*araBAD*)*567*). BW36931 samples were revived from frozen stocks on TYE agar, serially propagated on glucose M63 and glucose MOPS 2.0 mM Pi agar, then inoculated into 0.04% glucose MOPS 2.0 mM Pi broth and grown overnight before use, essentially as described elsewhere [26]. Portions were diluted into 0.4% glucose MOPS 2.0mM Pi broth and incubated at 37°C until the sample chamber was readied (ca. 40 min.).

To decrease the time delay between protein synthesis and fluorescence detection, we used the fast maturing Venus [27]. Tsr-Venus was synthesized at a low level under the control of the *phoA* promoter (*phoAp*) during growth in media with excess phosphate in which *phoAp* expression is turned off [28].

### Sample chamber preparation

Bottoms (18 mm diameter) of bottom glass fluorodish were immersed in pure sulphuric acid (H_2_SO_4_) for 5 min, washed in deionized water, sonicated in methanol and deionized water for 20 min, respectively. Residual liquid was dried with a stream of nitrogen gas. Cover glasses were coated with 100 μl of 1% polyethyleneimine (PEI) for 10 min. Cells were concentrated by gentle centrifugation in a minifuge (4,000 rpm, 1 min), resuspended, pipetted into the chamber and allowed to settle on the PEI layer for 20 min. Free floating cells were removed by washing surfaces with 0.4% glucose MOPS 2.0 mM Pi medium and 3 ml of fresh medium was added to the chamber prior to observation. Cell concentrations were adjusted so approximately 10 cells were visible in the field of view.

### Single molecule fluorescence video microscopy

The excitation laser (Argon ion, 488 nm emission, Newport) was expanded, filtered (488/10 nm line width bandpass filter, Chroma) and directed towards the microscope objective (100x, NA 1.4 oil immersion, Zeiss) parallel but off the optical axis through a dichroic mirror (500 nm Cutoff, Chroma). The resultant fluorescence image was projected through the dichroic mirror and an emission filter (525/50 nm bandpass, Chroma) and collected on a dual MCP intensified, cooled CCD camera (XR/Turbo-120z Turbo, Stanford Photonics, Inc.). The excitation beam was set such that it is just outside of the condition for total internal reflection, thus allowing for a deeper excitation (and deeper photobleaching) while still reducing background due to excess fluorescent matter in solution. For photobleaching steps, for the digitized illumination, the initial fluorescence synthesized overnight was completely photobleached by using 10-fold higher laser power (0.3 w/cm^2^ to 3 w/cm^2^). After 1 min without illumination, laser was turned on for 20 sec for data acquisition and turned off until next imaging slot was opened. This process was repeated every min 5 times. For continuous illumination, fluorescence imaging data were continuously acquired immediately after photobleaching.

### Recovery experiments

After acquiring prebleaching fluorescence images, the shutter on the microscopy (IX71 Inverted, Olympus) covered one pole while the other exposed to laser illumination. After one pole is completely photobleached, laser was turned off and shutter was removed and the fluorescence image was acquired for the shuttered pole.

All data files are available from the Purdue University Research Repository database, DOI: http://doi.org/10.4231/R7KK991C

## Supporting information

S1 File File contains: Supplemental Information I. Test of Non-Gaussianity, Supplemental Information II. Velocity Autocorrelation Function (VACF), Fig A: Tsr-Venus molecule dwells longer at the old pole. A) Ensemble fluorescence image of E. coli, predominately expressing Tsr-Venus molecules at the old pole. B) Combined single-image frame derived from four sequential movies [S1 Movie], showing new synthesis of individual Tsr-Venus molecules at both poles. Initial fluorescence signals in A were completely photobleached and newly made single Tsr-Venus molecules were detected. C) Intensity profiles of individual Tsr-Venus molecules as a function of its photobleaching time. Black and red arrows mark for old and young poles, respectively, and Table A: Number and location of newly synthesized Tsr-Venus proteins in one minute time intervals after complete photobleach (n = 1956 cells).
(PDF)

S1 Movie Photobleaching of pre-expressed Tsr-Venus molecules aggregated at both poles of E.coli. Similar fluorescence intensity at both poles at t = 0 was photobleached at different rates. The movie also shows that small clusters or single Tsr-Venus molecules are more mobile near the young pole.
(AVI)

S2 Movie Pre-existing Tsr-Venus molecules at the old pole (Fig A in S1 File) were photobleached prior to continuous illumination to detect newly synthesized Tsr-Venus molecules. The combined four sequential movies (cut from individual movies of the same cell) show single Tsr-Venus molecules that appear and disappear at both poles in a digitize manner. All Tsr-Venus disappeared several frames faster at the young pole (Fig A in S1 File).
(AVI)

S3 Movie Tsr-Venus molecules can escape from the old pole (bright) and diffuse throughout the cell to bind temporarily at the young pole. The corresponding spt-PALM is shown in the first panel of Fig 4C. Video rate is 30 fps.
(AVI)

S4 Movie Example of small clusters or single Tsr-Venus molecules that diffuse near the young pole with temporary binding as captured in the spt-PALM images of Fig 4C. (AVI)

S5 Movie Example of small clusters or single Tsr-Venus molecules that diffuse near the young pole with temporary binding as captured in the spt-PALM images of Fig 4C. (AVI)

S6 Movie Example of small clusters or single Tsr-Venus molecules that diffuse near the young pole with temporary binding as captured in the spt-PALM images of Fig 4C. (AVI)
