# Supplementary material for: Asymmetric polar localization dynamics of the serine chemoreceptor protein Tsr in Escherichia coli
Source: PLoS One. 2018 May 17;13(5):e0195887. doi: 10.1371/journal.pone.0195887 (PMC5957405; doi:10.1371/journal.pone.0195887)
Supplement: S1 File — Test of Non-Gaussianity, Supplemental Information II. Velocity Autocorrelation Function (VACF), Fig A: Tsr-Venus molecule dwells longer at the old pole. A) Ensemble fluorescence image of E. coli, predominately expressing Tsr-Venus molecules at the old pole. B) Combined single-image frame derived from four sequential movies [S1 Movie], showing new synthesis of individual Tsr-Venus molecules at both poles. Initial fluorescence signals in A were completely photobleached and newly made single Tsr-Venus molecules were detected. C) Intensity profiles of individual Tsr-Venus molecules as a function of its photobleaching time. Black and red arrows mark for old and young poles, respectively, and Table A: Number and location of newly synthesized Tsr-Venus proteins in one minute time intervals after complete photobleach (n = 1956 cells). (PDF) [file pone.0195887.s001.pdf]

Supplementary Information

Polar localization of serine chemoreceptor protein Tsr in Escherichia coli

Dongmyung Oh[1,†*], Yang Yu[2,‡], Hochan Lee[1], Jae-Hyung Jeon[3], Barry L Wanner[4*] and Ken Ritchie[1*]

[1]Department of Physics and Astronomy, Purdue University, 525 Northwestern Ave., West Lafayette, IN 47907, USA; [2]Department of Biological Sciences, Purdue University, West Lafayette, IN 47097, USA; [3]Department of Physics, Pohang University of Science and Technology, Pohang 02455, South Korea; [4]Department of Microbiology and Immunobiology, Harvard Medical School, Boston, MA 02115, USA

[†]Current address: Mechanobiology Institute, National University of Singapore, Singapore 117411, Singapore

[‡]Current address: Department of Preventive Medicine, Keck School of Medicine, University of Southern California, Los Angeles, CA 90033, USA

*To whom correspondence should be addressed: E-mail (K.R.) kpritchie@purdue.edu, (D.O.) mbiod@nus.edu.sg or (B.L.W.) Barry_Wanner@hms.harvard.edu

I. Test of Non-Gaussianity

We investigated whether the diffusion pattern of Tsr-Venus molecules follows an ordinary Gaussian behavior or non-Gaussian diffusion depending on time scales. A direct way of testing Gaussianity is to evaluate the van Hove self-correlation function

$$P(\boldsymbol{r},\Delta) = \langle \delta(\boldsymbol{r} - \boldsymbol{x}(t+\Delta) + \boldsymbol{x}(t)) \rangle_t,$$

which can be understood as the probability density function of finding the particle's displacement in [**x**, **x**+d**x**] for given lag time Δ. In d-dimensional space, Gaussian processes will have a van Hove self-correlation function of the form

$$P(\boldsymbol{r},\Delta) = \frac{1}{(4\pi D_\alpha \Delta^\alpha)^{d/2}} \exp\left(-\frac{\boldsymbol{r}^2}{4D_\alpha \Delta^\alpha}\right).$$

P describes the general case where the Gaussian process has a variance of

$\langle r^2(\Delta)\rangle = 2dD_\alpha\Delta^\alpha$ ($D_\alpha$is the generalized diffusivity and $0 < \alpha < 2$). Experimentally, the P profile can be distorted at the tail or totally blurred depending on the bin size due to the lack of sufficient data points.

To overcome this problem practically, it can be useful to examine the cumulative distribution of *P*.

$$\Pi(r^2,\Delta) = \int_0^r P(r,\Delta)2\pi r' dr'$$

$$=1- \exp\left(-\frac{r^2}{4D_\alpha\Delta^\alpha}\right) \text{ in } d=2.$$

Thus, we obtain the relation

$$-\ln(1-\Pi(r^2,\Delta)) \propto r^2$$

which means that the plot of $-\ln(1-\Pi)$ vs r^2 increases with slope 1 in log-log scale. If the slope deviates from 1, it signifies a non-Gaussian diffusion. It can be shown that when the van Hove function is non-Gaussian of the form

$$P(\boldsymbol{r},\Delta) \propto \exp\left[-\left(\frac{|\boldsymbol{r}|}{c\Delta^{\frac{\alpha}{2}}}\right)^\delta\right],$$

the cumulative distribution satisfies the scaling relation at large *r*[1].

$$-\ln(1-\Pi(r^2,\Delta)) \propto \left(r/c\Delta^{\frac{\alpha}{2}}\right)^\delta .$$

Experimental results

We calculated the cumulative distribution for the experimental data set; the lateral motion of Tsr molecules was recorded at various video frames to study their dynamic behavior at different time scales.

Tsr trajectories (1000 fps)

Figure S1 shows the cumulative distributions as a function of r^2 for lag times $\Delta = 1, 5$ ms. Both curves increase in a very similar fashion, with the scaling $\sim r^2$ expected from a Gaussian process. This behavior is consistent with our previous study[2]; at the shortest time

scale (of 1 ms), the Tsr-Venus molecule have no confining structure, thus, performing a normal unrestricted diffusion regardless of its location being at young or old pole.

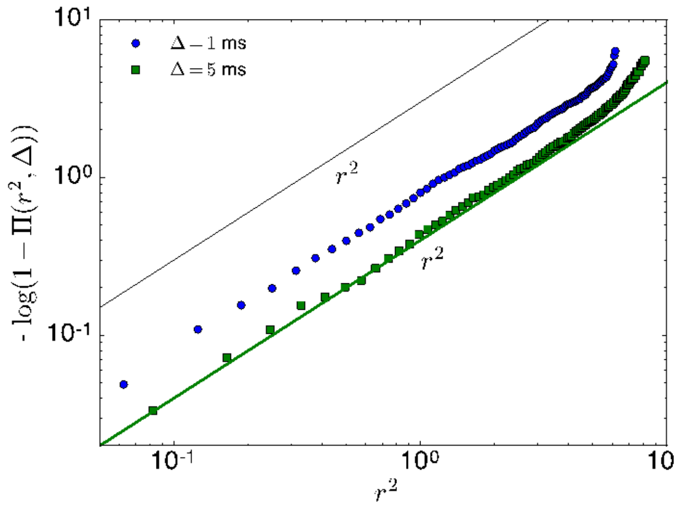

Figure S1 The cumulative distribution $-\ln(1-\Pi(r^2,\Delta))$ vs. r^2 for Tsr molecules observed at the video frame fps=1000. Two curves correspond to the results for $\Delta = 1, 5$ ms and the solid line depicts the scaling guide line of r^2.

Tsr trajectories (30 fps)

Figure S2 presents the cumulative distributions at lag times $\Delta = 33, 165, 330$ ms for Tsr-Venus molecules which were mobile at 30 fps. Here, the mobile Tsr-Venus molecules originate mostly from molecules diffusing at the young pole and partially from ones escaping from the old pole.

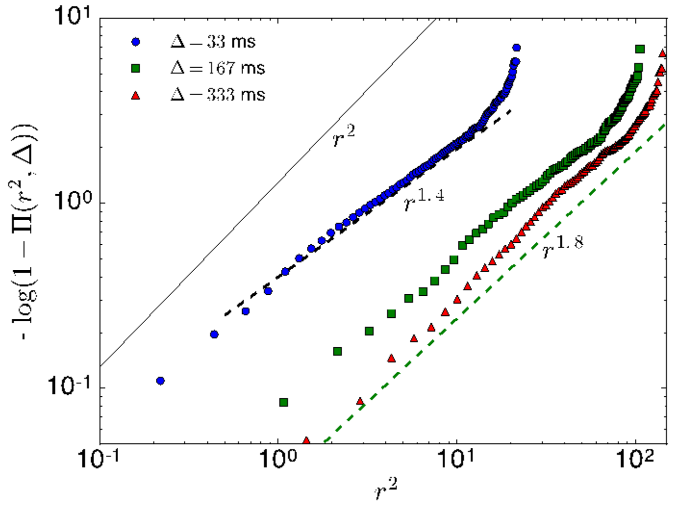

Figure S2 The cumulative distribution $-\ln(1-\Pi(r^2,\Delta))$ vs. r^2 for Tsr molecules observed at the video frame fps=30 and the lag times $\Delta = 33, 167, 333$ ms. Three scaling curves are depicted for guidance.

At times from tens to hundreds of ms, the cumulative distributions deviate significantly from the expected Gaussian behavior. Now $-\ln(1 - \Pi(r^2, \Delta))$ grows with *r* with the exponents [1.4…1.6]. This strong non-Gaussian character implies that lateral diffusion of Tsr-Venus is not simple Brownian. Rather, there appears to be multiple physical sources leading to the non-Gaussian behavior such as the curvature effect near the pole, binding-unbinding events with other proteins in the membrane, clustering-unclustering with other Tsr molecules, and others.

The van-Hove self-correlation function is plotted below (Fig. S3). Indeed, the *P* is highly non-Gaussian, having a cusp at the center.

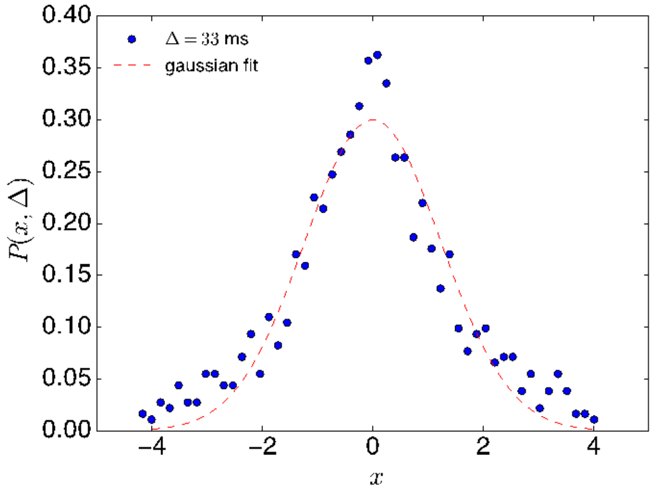

Figure S3 The van Hove self-correlation function $P(x, \Delta = 33\,ms)$ vs x. The dotted line represents a best Gaussian fit to the data.

We also found that the portion of Tsr molecules that were stationary during observation, which mostly originate from the old pole, has non-Gaussian character, albeit less (Data not shown). Further testing the non-Gaussianity of other data sets (400 - 60 fps) resulted in all non-Gaussianity except above 1000 fps data set, suggesting that molecules are not stably bound but rather are trapped in a cluster for certain time.

Simulation results

We performed Monte Carlo simulations on the diffusion of Tsr molecules on the surface of an *E. coli* cell. Details are described elsewhere[2]. This was done by modeling *E. coli* as a

cylinder of axial distance 2 μm with semi-spherical caps of radius 1 μm at both ends. The time step was chosen as 33 ms (30 fps) and the step length of 6.3 nm corresponding to an experimentally estimated diffusion constant $D = 0.002\ \mu m^2/s$. Although the simulation was performed on the curved surface, the position of a tracer was projected onto a reference flat frame and recorded for consistency with how experimental data were collected.

Tsr trajectories (30 fps)

Figure S4 shows the cumulative distributions in simulation at $\Delta = 33, 165, 330$ ms. Surprisingly, the normal diffusion on the curved surface results in non-Gaussian behavior similar to what was found experimentally at 30 fps; the exponent (slope) 1.7 lies in the values found in *in vivo* Tsr-Venus diffusion in Fig. S2. This finding supports the hypothesis that the membrane curvature at the poles of *E. coli* gives rise to nontrivial non-Gaussian effect on Tsr diffusion. However, the non-Gaussian behavior observed experimentally may not be solely attributed to the curvature effect. While the simulation results show the same scaling behaviors for all three lag times, the experimental curves have different scaling at different lag times, additional factors may play a role.

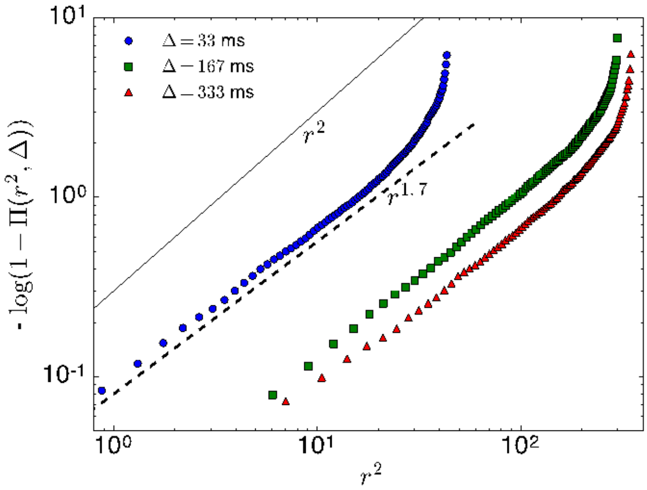

Figure S4 The cumulative distribution $-ln(1 - \Pi(r^2, \Delta))$ vs. r^2 in simulation for the lag times $\Delta = 33,167,333$ ms. Two scaling curves are depicted for guidance.

II. Velocity autocorrelation function (VACF)

We define the average velocity of a Tsr molecule as $\boldsymbol{v}(t;\delta t) = [\boldsymbol{r}(t+\delta t) - \boldsymbol{r}(t)]/\delta t$ for an arbitrary time interval δt. In the limit of $\delta t \to 0$, the average velocity $\boldsymbol{v}(t;\delta t)$ becomes the instantaneous velocity $\boldsymbol{v}(t)$, but in experimental situations (i.e., the overdamped limit) the $\boldsymbol{v}(t)$ is unattainable and only the average velocity $\boldsymbol{v}(t;\delta t)$ can be defined via the positional information. In this sense, $\boldsymbol{v}(t;\delta t)$ can be understood as displacement correlation for given time interval, e.g., $\delta t = \Delta t_0$(video frame interval), $5\Delta t_0, 10\Delta t_0$, etc.

With the $\boldsymbol{v}(t;\delta t)$, VACF is defined in the level of a single trajectory $\boldsymbol{r}^i(t)$ as

$$C_v^{(i)}(\Delta) = \langle \boldsymbol{v}(t+\Delta;\delta t) \cdot \boldsymbol{v}(t;\delta t) \rangle_t$$

where $\langle \ldots \rangle_t$ means the average along the trajectory and Δ is the lag time. The normalized VACF is $C_v^{(i)}(\Delta)/C_v^{(i)}(0)$. For N trajectories, the average over all particles is defined as

$$C_v(\Delta) = \sum_{i=1}^{N} C_v^i(\Delta)/N.$$

We plot this averaged VACF curve below.

Experimental results

Tsr trajectories (1000 fps)

Figure S5 shows the VACF curves for time intervals $\delta t = \Delta t_0$(=1 ms, the video frame interval) and $5\Delta t_0 = 5$ ms. Both curves behave similarly; there is a dip at $\Delta = \delta t$ and a fast relaxation towards zero for $\Delta > \delta t$. The position of the dip changes with δt because any two average velocities $\boldsymbol{v}(t_1;\delta t)$ and $\boldsymbol{v}(t_2;\delta t)$ are positively correlated if $|t_1 - t_2| < \delta t$, in accordance with the definition of $\boldsymbol{v}(t;\delta t)$. The dips signify an anti-persistent correlation in Tsr motion at time scales ~ O(1 ms). However, its duration is merely of δt and, accordingly, Tsr molecules perform a memoryless random walk, which is consistent with the Gaussian behavior at this time scale in the cumulative distribution (Fig. S1).

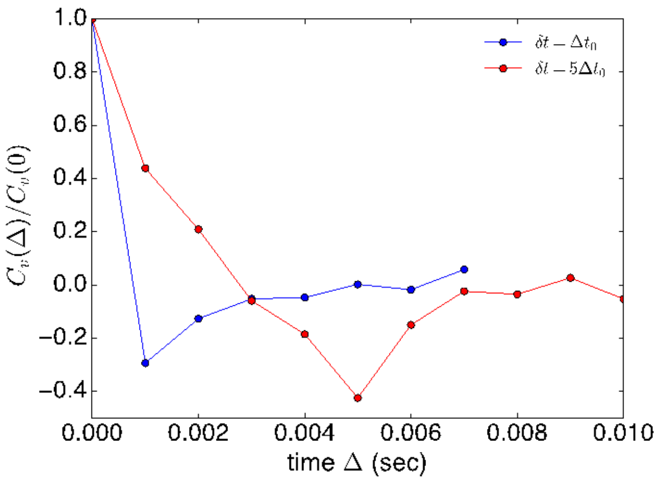

Figure S5 The normalized VACF curves for the average velocities defined with $\delta t = \Delta t_0 = 1$ *ms and* $5\Delta t_0$.

Tsr trajectories (30 fps)

Figure S6 shows the VACF curves for mobile Tsr molecules at time intervals $\delta t = \Delta t_0$=33 ms and $5\Delta t_0 = 167$ ms. The motion appears to be an ordinary random walk including a short-lived negative correlation when observed at the interval of 33 ms. However, at longer intervals, the VACF exhibits an oscillating decay pattern, which was obtained from 3 long trajectories containing > 50 data points. Individual VACF curves have consistent oscillatory patterns (Fig. S7). With the non-Gaussianity character (Fig. S2), the oscillating VACF suggests that Tsr long-distance motion of Tsr *in vivo* has a more complicated diffusion associated with the cell geometry, membrane crowding, and clustering.

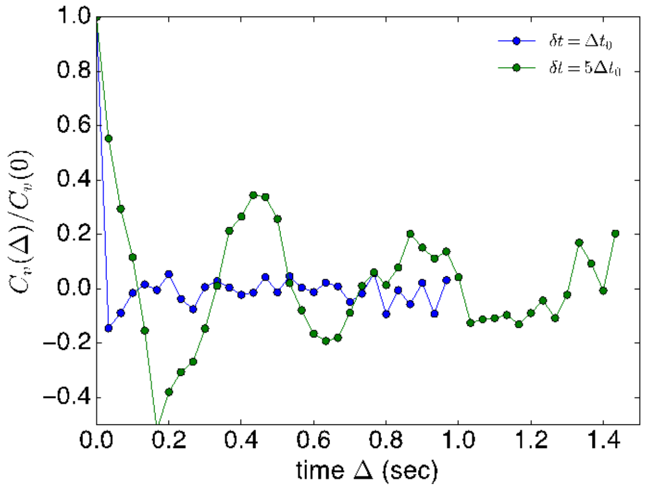

Figure S6 The normalized VACF curves for the average velocities defined with $\delta t = \Delta t_0 = 33$ ms and $5\Delta t_0 = 167$ ms.

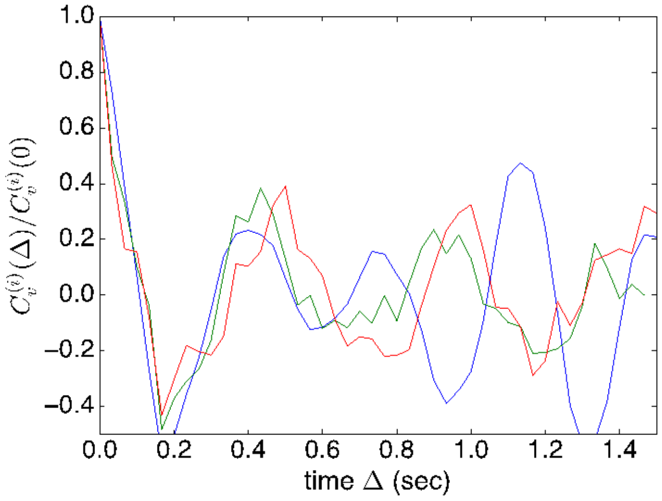

Figure S7 The three individual VACF curves for the average VACF curve in Fig. S6 for $\delta t = 5\Delta t_0$.

Simulation results

Tsr trajectories (30 fps)

Figure S8 presents the VACF curves for the trajectories from simulation. The result for $\delta t = \Delta t_0$ is similar to the experimental one. However, the curve for $\delta t = 5\Delta t_0$ has no oscillation in the experiment, which means that the oscillation is not likely the consequence from the membrane curvature, albeit it gives rise to the non-Gaussian character in the observed Tsr motion (Fig. S6).

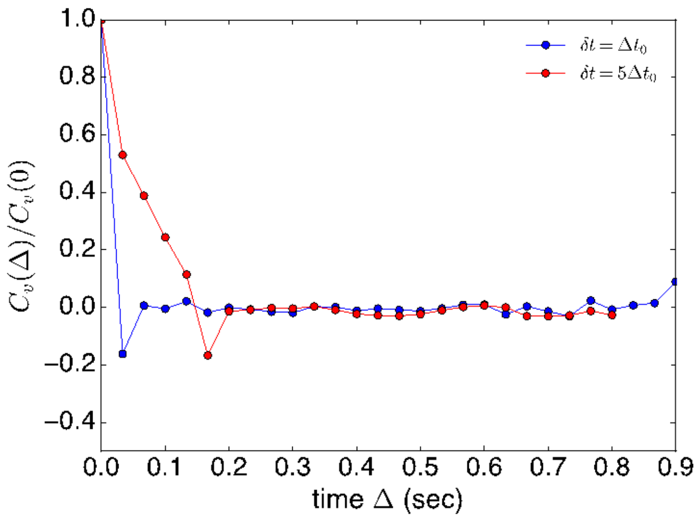

Figure S8 The normalized VACF curves in simulation for $\delta t = \Delta t_0 = 33$ ms and $5\Delta t_0 = 167$ ms.

Additional Supplement Figures and Movies

Supplemental Figure S9. Tsr-Venus molecule dwells longer at the old pole. A) Ensemble fluorescence image of E. coli, predominately expressing Tsr-Venus molecules at the old pole. B) Combined single-image frame derived from four sequential movies [Supplement movie 1], showing new synthesis of individual Tsr-Venus molecules at both poles. Initial fluorescence signals in A were completely photobleached and newly made single Tsr-Venus molecules were detected. C) Intensity profiles of individual Tsr-Venus molecules as a function of its photobleaching time. Black and red arrows mark for old and young poles, respectively.

Movie 1: Photobleaching of pre-expressed Tsr-Venus molecules aggregated at both poles of E.coli. Similar fluorescence intensity at both poles at t=0 was photobleached at different rates. The movie also shows that small clusters or single Tsr-Venus molecules are more mobile near the young pole.

Movie 2 (for Supplement Fig 1): Pre-existing Tsr-Venus molecules at the old pole (Supplement Fig 1) were photobleached prior to continuous illumination to detect newly synthesized Tsr-Venus molecules. The combined four sequential movies (cut from individual movies of the same cell) show single Tsr-Venus molecules that appear and disappear at both

poles in a digitize manner. All Tsr-Venus disappeared several frames faster at the young pole (Supplement Figure 1).

Movie 3: Tsr-Venus molecules can escape from the old pole (bright) and diffuse throughout the cell to bind temporarily at the young pole. The corresponding spt-PALM is shown in the first panel of Fig 4C. Video rate is 30 fps.

Movies 4, 5, and 6: Examples of small clusters or single Tsr-Venus molecules that diffuse near the young pole with temporary binding as captured in the spt-PALM images of Fig 4C.

1 Jeon, J.-H., Javanainen, M., Martinez-Seara, H., Metzler, R. & Vattulainen, I. Protein crowding in lipid bilayers gives rise to non-gaussian anomalous lateral diffusion of phospholipids and proteins. *Phys.Rev.* **X6**, 021006 (2016).

2 Oh, D., Yu, Y., Lee, H., Wanner, B. L. & Ritchie, K. Dynamics of the serine chemoreceptor in the Escherichia coli inner membrane: a high-speed single-molecule tracking study. *Biophys J* **106**, 145-153, doi:10.1016/j.bpj.2013.09.059 (2014).

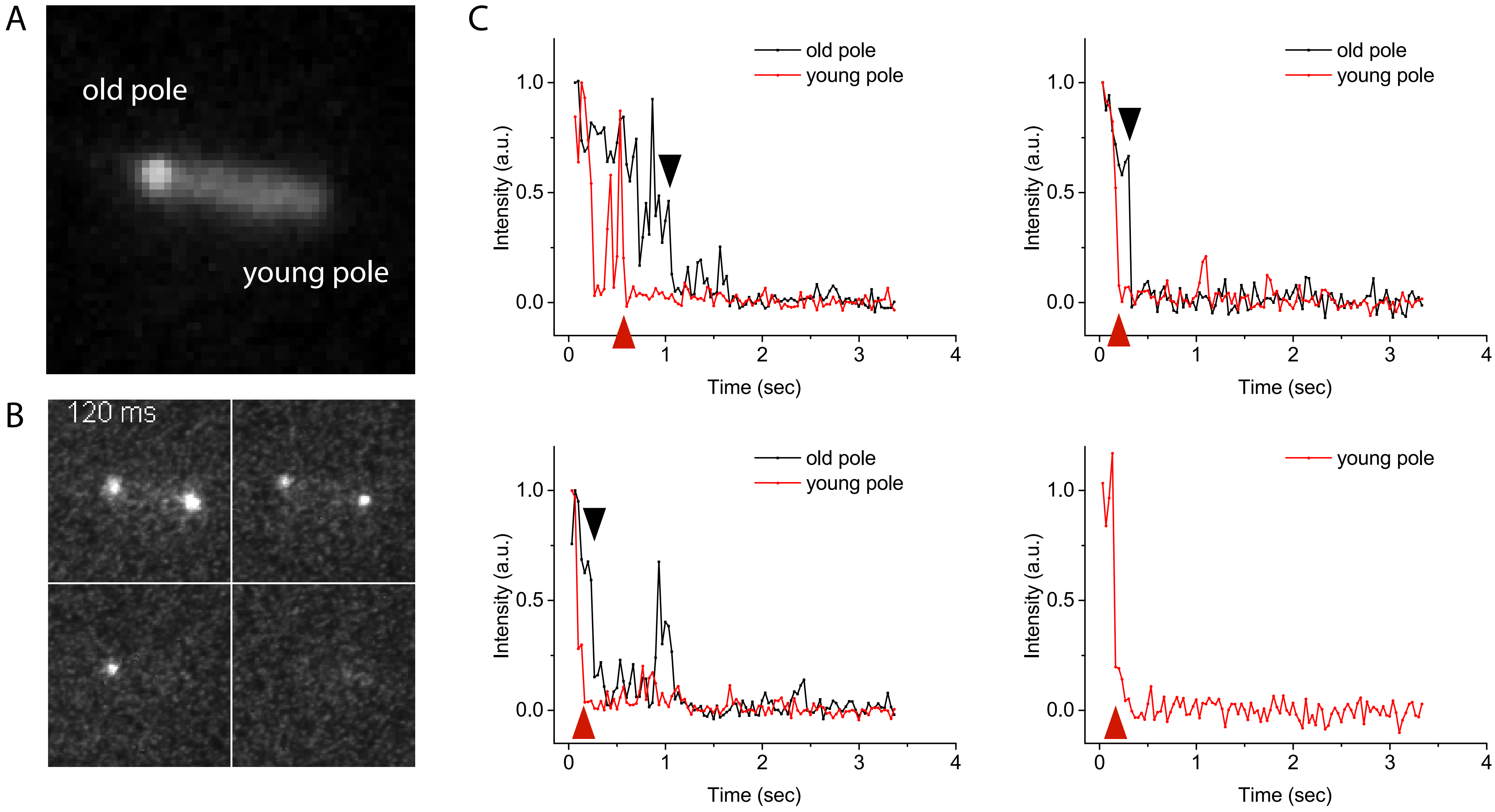
A
old pole
young pole
B
120 ms
C
old pole
young pole
Intensity (a.u.)
Time (sec)
1.0
0.5
0.0
0
1
2
3
4

Figure A

Table A. Number and location of newly synthesized Tsr-Venus proteins in one minute time intervals after complete photobleach (n = 1956 cells)

Recovery Time	# expressed at poles	# expressed in central region	Accumulated total
1 min	1176	230	1406
2 min	375	67	1848
3 min	273	57	2178
4 min	70	38	2286
5 min	41	29	2356
